# Complete Genome Sequence of a Listeria monocytogenes Strain with Antimicrobial Resistance Genes Isolated from Lettuce in Canada

**DOI:** 10.1128/mra.00298-22

**Published:** 2022-06-06

**Authors:** Amit Mathews, Sohail Naushad, Marc-Olivier Duceppe, Mingsong Kang, Lin Ru Wang, Hongsheng Huang

**Affiliations:** a Ottawa Laboratory-Fallowfield, Canadian Food Inspection Agency, Ottawa, Ontario, Canada; b Greater Toronto Area Laboratory, Canadian Food Inspection Agency, Toronto, Ontario, Canada; University of Maryland School of Medicine

## Abstract

Listeria monocytogenes, a Gram-positive, rod-shaped, non-spore-forming bacterium, is an important foodborne bacterial pathogen for humans worldwide. Here, we report the complete genome sequence of a Canadian Listeria monocytogenes strain with an antimicrobial resistance (AMR) gene that was isolated from lettuce.

## ANNOUNCEMENT

Listeria monocytogenes, a Gram-positive bacterium that is found in various environments, animals, and food, is one of the most important foodborne pathogens for humans worldwide due to its high mortality rate (1). We report the complete genome sequence of L. monocytogenes strain GTA-L258, which was isolated from lettuce taken in Canada in 2015. The strain was isolated by preenrichment in *Listeria* enrichment broth at 30°C for 24 h, selective enrichment in buffered *Listeria* enrichment broth with morpholinepropanesulfonic acid (MOPS) (MOPS-BLEB) at 35°C for 18 to 24 h, and isolation from preenrichment cells grown on Oxford agar at 35°C for 48 h and on RAPID'*L.mono* (RLM) medium at 35°C for 24 h, with confirmation by hemolysis on blood agar plates at 35°C for 24 h, motility testing on tryptose agar at 30°C for 24 h, and Vitek (bioMérieux, Canada) testing (2).

Genomic DNA (gDNA) was extracted from an overnight culture in brain heart infusion medium using a Maxwell 16-cell DNA purification kit (Promega, USA) for Illumina sequencing and a NanoBind CBB Big DNA kit (Circulomics, USA) for Nanopore sequencing and was quantified using a Qubit fluorometer (Thermo Fisher Scientific, USA). MiSeq sequencing was conducted by library preparation with the Nextera XT library preparation kit (Illumina, USA) followed by sequencing for 600-bp cycles, which produced 576,050 paired-end reads with 89× coverage. Nanopore sequencing was performed using the 1D native barcoding gDNA protocol (EXP-NBD104 and SQK-LSK109; Oxford Nanopore Technologies, UK) without shearing, followed by sequencing using a FLO-MIN106 (R9.4.1) flow cell on a MinION MK1C device, which produced 115,064 reads (average length, 15,598 bp; *N*_50_, 35.6 kb) with 466× coverage. Base calling was performed using Guppy v5.0.11 in super high accuracy mode, trimming using Porechop v0.2.3 (3), and filtration using Filtlong v0.2.1 (4). Assembly of the long reads was performed using Flye v2.7 (5), corrected using Medaka v1.4.4, and polished with Illumina MiSeq reads using a combination of NextPolish v1.4.0, ntEdit v1.3.5, and Polypolish v0.5.0 after trimming and filtering with fastp v0.23.2. The circularity and genome rotation using *dnaA* as the starting point were determined using the fixstart plugin from Circlator v1.5.5 (6). The sequencing coverage depth was determined using minimap2 v2.17 (7) and SAMtools v1.13 (8) for long reads and BWA v0.7.17 and SAMtools v1.13 for short reads. Gene prediction and annotation were performed using the NCBI Prokaryotic Genome Annotation Pipeline (PGAP) v6.0 (9).

Antimicrobial resistance (AMR) genes were identified using ResFinder v4.1.5 (10) and RGI v5.2.0 (11). The plasmids were identified by mlplasmids v1.0.0 using Listeria monocytogenes as the species model (12). Prophage sequences were analyzed using PHASTER (13) (accessed online on 31 January 2022). Default parameters for pipelines were used except where otherwise noted (https://github.com/OLF-Bioinformatics/nanopore).

The GTA-L258 complete genome contains a single chromosome (2,939,920 bp) harboring a *fosX* AMR gene, without identification of plasmids or intact prophages (PHASTER score of >90). The annotated genome contains 2,839 coding sequences (CDSs) and 67 tRNAs, with a genomic GC content of 37.89%, similar to 2,889 CDSs and a GC content of 37.88% on average for L. monocytogenes strains in the NCBI database (accessed 31 January 2022).

### Data availability.

The whole-genome sequence of GTA-L258 was deposited in GenBank under accession number CP092060. MinION base-called and MiSeq base-called fastq files are available in the NCBI Sequence Read Archive (SRA) under accession numbers SRR17965225 and SRR17965217, respectively.
